# Automatic patient positioning based on robot rotational workspace for extended reality

**DOI:** 10.1007/s11548-023-02967-2

**Published:** 2023-06-09

**Authors:** Marek Żelechowski, Balázs Faludi, Murali Karnam, Nicolas Gerig, Georg Rauter, Philippe C. Cattin

**Affiliations:** 1 Center for medical Image Analysis & Navigation (CIAN), Department of Biomedical Engineering, University of Basel, Basel, Switzerland; 2 Bio-Inspired RObots for MEDicine-Laboratory (BIROMED-lab), Department of Biomedical Engineering, University of Basel, Basel, Switzerland

**Keywords:** Computer-assisted surgery, Patient positioning, Augmented reality

## Abstract

**Purpose:**

Understanding the properties and aspects of the robotic system is essential to a successful medical intervention, as different capabilities and limits characterize each. Robot positioning is a crucial step in the surgical setup that ensures proper reachability to the desired port locations and facilitates docking procedures. This very demanding task requires much experience to master, especially with multiple trocars, increasing the barrier of entry for surgeons in training.

**Methods:**

Previously, we demonstrated an Augmented Reality-based system to visualize the rotational workspace of the robotic system and proved it helps the surgical staff to optimize patient positioning for single-port interventions. In this work, we implemented a new algorithm to allow for an automatic, real-time robotic arm positioning for multiple ports.

**Results:**

Our system, based on the rotational workspace data of the robotic arm and the set of trocar locations, can calculate the optimal position of the robotic arm in milliseconds for the positional and in seconds for the rotational workspace in virtual and augmented reality setups.

**Conclusions:**

Following the previous work, we extended our system to support multiple ports to cover a broader range of surgical procedures and introduced the automatic positioning component. Our solution can decrease the surgical setup time and eliminate the need to repositioning the robot mid-procedure and is suitable both for the preoperative planning step using VR and in the operating room—running on an AR headset.

## Introduction

Robot-assisted surgery (RAS) is becoming increasingly common as technology advances; the conviction about the safety and effectiveness of robots prevails [10, 26]. A robotic arm allows surgeons to operate with greater precision and control and is often less invasive than traditional, open surgery. This results in less pain and scarring for the patient, shorter hospital stays, and a quicker recovery.

Many systems today select a master-agent configuration where the surgeon controls the instruments using a computer console that gives him/her a 3D image of the surgical site with a robotic manipulator beside the patient on a mobile cart, e.g., da Vinci Xi, Intuitive Surgical, USA. Having a patient cart introduces a level of flexibility in an already cluttered operating room (OR) and allows the system to be employed for different surgical procedures. At the same time, this introduces extra work for the staff to prepare the draping, positioning, and docking of the robot [4, 24].

Patient positioning is a critical component of robotic surgery, which affects both the technical and medical side of the procedure. There are various ways to position a patient for robotic surgery, and the best way to arrange a patient will vary depending on the surgery being performed. A proper setup minimizes the risk of potential complications, such as nerve damage or blood clots [17], and provides optimal mobility for the robotic end-effector [20]. Apart from good access, the patient should be positioned in a way that allows for proper monitoring during the procedure. These factors improve the range of motion (RoM) available to the surgeon and streamline the procedure. All these factors emphasize the need for a preoperative planning step.

Preoperative planning is important for robotic surgery because it helps to ensure the success of the procedure and reduce the risk of complications. During this stage, the surgeon reviews the patient’s medical history and imaging data, such as computer tomography (CT) or magnetic resonance imaging (MRI) scans, to get a better understanding of the patient’s anatomy. The surgeon then develops a surgical plan, taking into account the characteristics of the surgical robot. Having access to the rotational workspace data of the robotic arm in this steps ensures that the robotic arm can be positioned in the most optimal way, removing the need to reposition and redock it mid-procedure.

Single-port laparoscopy (SPL) is a relatively new development in minimally invasive interventions, where the surgeon inserts a small camera and surgical instruments through a single incision. This type of surgery is associated with less pain and scarring and faster recovery time than more traditional multi-port laparoscopy (MPL). At the same time, SPL is considered a more demanding approach, not suitable for every intervention as it largely depends on the available working space [19]. With the introduction of systems like da Vinci SP, single-port interventions still lag behind the operations performed using multi-port robotic systems [13, 29].

Recently, general purpose lightweight robots are used for medicine and surgery, for example the KUKA LBR iiwa med (KUKA A.G., Germany) to assist ultrasound imaging [33] and to cut bones [1]. Such robots are safe for direct physical interaction and have the flexibility to mount different tools that are needed during surgery. However, the workspace of such robots is limited and understanding these workspace limits is important such that the surgeon can reach the desired locations without the need to setup the robot and/or patient multiple times during the intervention. In minimally invasive surgeries, the tools typically enter the patient through a trocar and the robot is constrained to move with a remote center (RCM); visualizing the rotational workspace limits at the remote center would be useful to plan the surgery and position the patient/robot. For industrial applications, methods have been developed to calculate and visualize the robot workspace with different maps, such as the capability map [34], IK-MAP [31] and reachability map [21]. The 6D workspace of the robot is typically visualized with these maps on a 2D screen, which is not completely intuitive for surgeons to inspect and understand it. At the same time, introducing a new robotic platform into the OR is challenging and requires a lot of training [16, 28]. Understanding the capabilities and limitations of the system takes time and can significantly prolong adoption time. Visualizing the robot’s workspace and automatically calculating the optimal patient location with respect to the robot could greatly aid the surgeon in positioning the patient before the surgery.

**Fig. 1:**
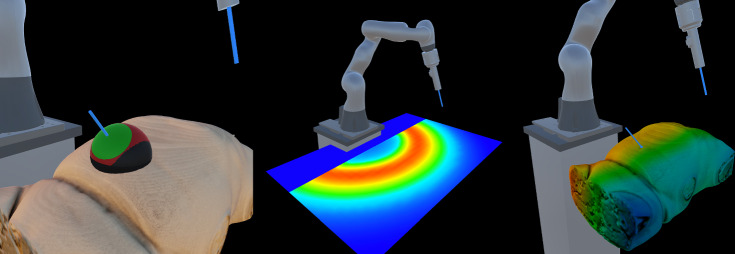
Three representations of the reachability map of the robot. The 5D map can be shown as a set of spheres, with the red patches indicating the reachable orientation of the endoscope while the tip is at the center (left). The dimensionality-reduced reachability map is shown overlaid on a plane (center) and on overlaid on the volumetric rendering of the preoperative patient data (right)

Virtual and augmented reality (VR/AR) find increasingly more interest in the medical field [11, 25]. They are used as a training tools [6, 23], in diagnostics [27, 32], and to educate patients about their condition [12]. The advancements in GPU technology and the development of high-resolution head-mounted displays enable a realistic visualization of preoperative imaging data utilizing direct volume rendering [9, 18]. Although computationally intensive, volume rendering allows visualizing 3D medical datasets without the time-consuming segmentation step, offering a much more flexible and faster way of examining the data [7, 35]. Transfer functions, which transform Hounsfield unit values into color and transparency and the possibility to change them dynamically, can considerably reduce the time between the image acquisition and the 3D visualization. Systems like SpectoVR can be used for diagnostics and surgical planning in an immersive VR environment [5]. At the same time, these systems deployed to AR headsets can visualize the same data and offer similar features in the OR.

Within the scope of the previous work, we demonstrated the benefits of using augmented reality in patient positioning tasks [36]. The users were tasked with positioning the virtual patient while being able to preview the robot’s rotational workspace using the visualization modes (see Fig. 1). In a patient positioning task, the users performed significantly better provided with workspace information displayed on AR glasses. At the same time, on average, they could not achieve the best possible results for a given robot’s configuration and trocar location.

This work builds upon the previous system and improves its capabilities and overall performance [36]. Based on the user results and feedback, we redesigned the system to automatically calculate the best robot-patient configuration using the robot’s rotational workspace and trocar locations. We increased the number of trocar locations taken into account when calculating the optimal patient position. The proposed improvements fit well into the requirements of a modern OR and simplify the inception of new robotic systems in the workflow. We carried out an extended performance analysis in different scenarios to assess the applicability of our system. In all tested scenarios, we reached performance levels that offer solutions in milliseconds range for the surface coverage metric and within seconds for the cone angle metric.

## Methods

The methods section describes the base implementation of each component and the changes made to the previously developed version of the system. In “Workspace calculation” section, we explain the process of calculating the rotational workspace for the employed robot configuration. “XR visualization” section covers the development of a port-placement plugin for the SpectoVR surgical planning tool and its integration with the main VR application. “XR visualization” section describes the modifications made to address the operating room (OR) requirements in the AR version of the system. “XR visualization” section discusses the existing methods for visualizing a robot’s workspace and the modes of presenting the relative patient-robot transform. Finally, in “Performance testing” section, we describe the validation procedure and the metrics used to evaluate the proposed port placement and performance.

### Workspace calculation

We used the forward kinematics approach [21] to calculate the robot’s 5D reachability map (3D position and 2D orientation) with an axially symmetric tool. The workspace bounds for the robot, a volume of ${(3\times 1\times 1.5\,{m^{3}}) \times (\pi \times \pi /2\,{rad})}$, were divided into 5D voxels of size 5${cm^{3}}$ in position and ${2}{^\circ }$ in orientation. A Boolean array of the workspace was created for each voxel, where true represented that the robot can reach that combination of position and orientation. The reachability map was calculated as a one-time task for the robot by iterating through the joint positions and setting the voxel closest to the tool’s pose as true. A minimum joint step of ${0.01}{^\circ }$ was used. To speed up the calculation, based on the current robot pose, the joint step was dynamically updated such that the robot pose was incremented to the next voxel. The calculation of the reachability map took 10hours to evaluate on a workstation (Intel Xeon CPU E6-2620 v4, 48GB RAM) using 16 threads in parallel for the KUKA LBR iiwa robot. The reachability map was reduced to a 3D map by calculating the percentage of the hemisphere’s area reachable by the robot to represent the orientation workspace at each position.

### XR visualization

Following the feedback from the surgeons, we decided to split our solution into two separate applications—the first offering an immersive VR experience for surgical planning and the second being a lightweight system to be used in the operating room. This division aims to address the different characteristics of the OR workflow, with time sensitivity at the center.

#### Virtual reality surgical planning

Specto is an extended reality (XR) medical data visualization software developed initially at the Department of Biomedical Engineering. The application was developed using the Unity engine (Unity Technologies Inc., USA), using direct volume rendering. This application can visualize any CT or MRI volumetric dataset. Implementing OpenXR (Khronos Group Inc., USA) provides support for most VR and AR platforms and devices conforming with the standard.

Recent changes introduced a plug-in system, where developers can develop extra features and easily integrate them into the core application. Utilizing this framework, we could extend the surgical planning feature set of SpectoVR with a trocar placement tool and an automatic patient/robot positioning system. Therefore, after examining the medical dataset, the user can select suitable port locations according to the target location and the robot’s specifications (see Fig. 3). The trocar is represented by a blue sphere with a cylinder indicating its orientation, and it can be positioned using a VR controller. Once the port positioning process is finished, the user triggers the optimal pose calculation. The results are then saved to a file, which the VR and AR applications can load.

#### AR application

Considering the workflow and the time-sensitive nature of the OR, we decided to deploy a stripped-down version of our software for AR glasses. The computational power of the ML1 (Magic Leap 1) headset is insufficient to allow for volume rendering of medical datasets and maintain 60 frames per second rendering performance. Specto offers an option to generate a mesh model from the volumetric dataset based on a given transfer function. This feature aims to address this exact issue, to allow low-powered devices to visualize the preoperative plan made on a more computationally capable machine.

**Fig. 2:**
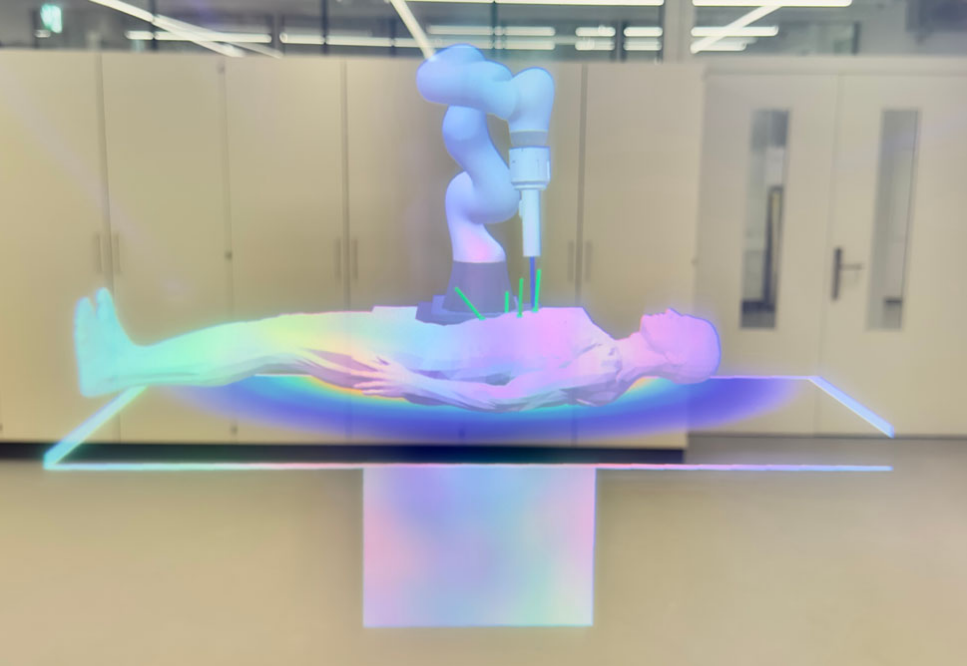
A photograph taken through the right lens of Magic Leap 1 headset. It shows a patient’s mesh model with 4 defined trocar positions and a calculated optimal location of the robotic arm. The robotic arm was set to a random joint configuration

The robot’s workspace data file is manually placed in the memory of the ML1 device. The AR application allows users to register the robot’s position in the OR using built-in image recognition. Then, they can examine the robot’s workspace using one of the visualization tools described in “XR visualization” section. The optimal robot/patient poses can then be loaded from the binary files and as visualized in AR (see Fig. 2). That said, it is still possible to reposition the trocars and trigger the recalculation of the optimal pose using the ML1 device. Due to the nature of the OR, the controller manipulations are replaced with a hand interaction system, which allows the user to move desired port locations using simple gestures and hand tracking.

#### Workspace representation

The workspace data loading remained primarily unchanged in comparison with the previous implementation. The 5D workspace map calculated as described in “Workspace calculation" Section is read from a binary file during the application startup phase. One noticeable change was the parallelization of the code, which reduced the loading times from 15 to 2 s. We maintained all the modes of workspace visualization in the VR module; therefore, 5D representations using a sphere and dimensionality-reduced 3D workspace can be visualized on top of any mesh or voxel object in the scene.

The workspace visualization sphere maintained the previous design. The surface of the sphere illustrates the rotational reachability of the robot at the center of the orb, with red areas indicating the direction from which the end-effector can reach the center position. The user can freely move the sphere around the scene using the controller, scanning the workspace for ideal trocar positions and finding a good patient location. Alternatively, the orb can be permanently attached to the trocar (see the left side of Fig. 1), moving together with the virtual patient and updating the texture based on the relative position of the robot. In this mode, the green circle is shown on the sphere, representing the maximal RoM available in any direction of roll-yaw at this location.

Similar to the previous work, we offer the user an intuitive preview of the 5D workspace reduced to a 3D texture. We represent the entire 2D orientation workspace visible on the sphere as a single number, the percentage of the hemisphere’s area reachable for the robotic arm. This value is then mapped to a predefined color gradient, creating a material that can be applied to any mesh or voxel object using a custom shader (see middle and right side of Fig. 1, respectively). This feature allows the user to quickly scan the volume to understand the available workspace of the robot.

**Fig. 3:**
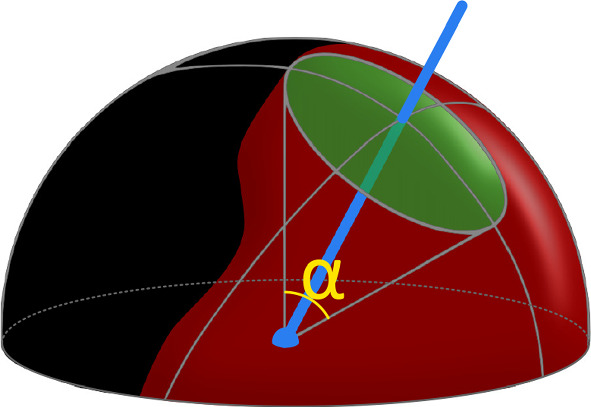
Graphical representation of the rotational sphere showing reachable orientations (red patches) at a given location. The green circle is indicating the maximal range of motion possible in any direction in a circular pattern; it is evaluated by finding the largest circle that can be drawn about the desired trocar direction (indicated by the blue line). The angle alpha describes the apex angle of the resulting cone

### Optimal pose calculation

Our previous implementation relied solely on the users to place the patient/robot in the correct location, aiding them by visualizing the workspace in the AR glasses. We introduced two metrics to evaluate a user’s performance: the hemisphere surface coverage in percentages and the cone angle in degrees. The surface area served to quantify the total orientation change around a given point. It was calculated as a percentage of the area of the hemisphere reachable by the robot at a given position in the workspace (red region of the hemisphere in Fig. 3). In turn, the cone angle indicates the greatest possible rotation in any direction of the central trocar axis. Figure 3 shows the cone angle as the apex angle of the created cone, with values ranging from 0 to 180$^{\circ }$.

We used these metrics to evaluate the performance of the user tasked with placing the patient at the optimal pose for a given trocar configuration. In order to establish the baseline for comparison between available visualization modes and users, we iterated through the entire workspace calculating both metrics at each position. Since it was a one-time computation, the performance was not a priority, resulting in a process taking around 90 min and being limited to a single port location.

**Fig. 4:**
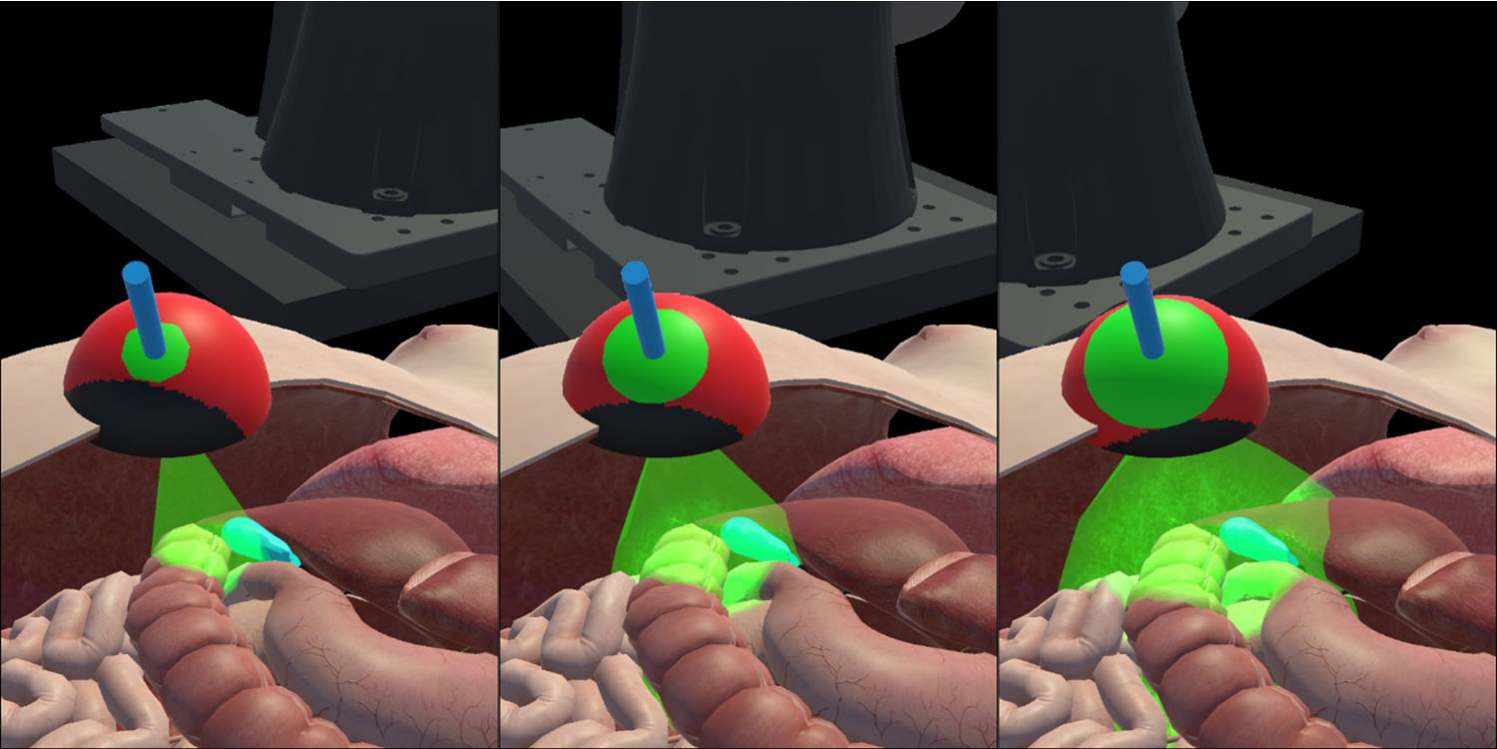
Three consecutive steps of our automatic positioning algorithm showcase the improvement in the rotational workspace at the trocar location for a single-port cholecystectomy. The patient’s/robot’s model is moved or rotated incrementally with each iteration to reach the optimal pose. Each section displays the abdominal cavity with a blue target (gallbladder), the rotational sphere (refer to Fig. 3), and a green semitransparent cone, representing the angular range of motion within the patient’s body

Due to the characteristics of the rotational workspace of our robotic system, we implemented a local search algorithm to find the optimal pose for a given trocar configuration. The algorithm begins by selecting a random starting point in the middle of the 3D workspace. It then evaluates the value of the metric for each of the neighboring points (including rotation around Y-axis). In subsequent iterations, the algorithm moves to the neighboring point with the highest value and repeats the process until no further improvement can be made. This approach allows the algorithm to efficiently search for and identify optimal patient-robot configuration (as visualized in Fig. 4). In combination with the parallelization of the code and other code optimizations, we drastically reduced the time required to find the solution.

Once the solution is found, the application presents a suggested robot/patient location for the user to evaluate it, and if deemed necessary, can be manually discarded, which triggers the recalculation of the algorithm omitting the deleted pose. Any consecutive runs utilize the information obtained during the already performed calculations. Therefore, the local search algorithm starts at the index selected previously as an optimal one, and whenever possible, it recycles the calculated metrics from previous steps, saving computational resources.

### Performance testing

Within the scope of the previous work, we showed how augmented reality can improve the results in the patient positioning task [36]. At the same time, on average, they could not achieve the best possible results for a given configuration. Therefore, after focusing on our automatic pose calculation algorithm, we ran a performance analysis for different scenarios to access the impact of our changes and improvements. The running times would allow us to decide if it is feasible to maintain all the functionalities in both versions of the application: VR running on a desktop PC, and standalone deployed to the AR glasses.

#### Hardware setup

Our system consists of two independent programs: (1) *desktop VR software for preoperative planning* and (2) *standalone AR visualization application suitable for the OR*. The desktop application ran on a Desktop PC (AMD Ryzen 3700X CPU, 32 GB DDR4 memory, and AMD Radeon 6800 16 GB GPU). We used an HP Mixed Reality VR headset for visualization and a 6 degrees of freedom (DoF) controller for the port placement in VR.

The AR application was deployed as a standalone build and ran on ML1 headset (Nvidia Parker SOC CPU, Nvidia Pascal GPU, and 8 GB RAM). This AR system is also equipped with a 6DoF controller that allows the user to interact precisely with the virtual objects located in the scene.

#### Testing scenarios

We selected a total of eight surgical scenarios with varying number of required ports. Single trocar: (a) at the umbilicus, 40$^{\circ }$ off the sagittal axis, a commonly used location in SPL [2, 13], (b) on the mid-clavicular line, typical in liver resection surgery [30]. Two ports: to account for the cases when a secondary incision is made during the SPL (for suction or retraction [14]). Initial port locations are the same as for the single case, with the second opening located 5cm to the right. Three ports: one set of the incisions corresponding to laparoscopic appendectomy [22] and a second used for cholecystectomy [3]. Four trocars: (a) cholecystectomy [15] and (b) total laparoscopic hysterectomy [8].

In each scenario, we covered multiple events that might occur in regular use of the software—rerunning the algorithm after one trocar was repositioned or excluding one of the proposed poses. For each case, we report the computation time needed to find an optimal patient/robot position by our algorithm on both devices: desktop VR setup and standalone AR headset. Additionally, we measured the time of calculating the optimal pose in a scenario when the trocar location has to be modified in the OR. Therefore, we compare the ML1 AR headset processing time for the first algorithm run and for consecutive runs where one random trocar was repositioned.

**Fig. 5:**
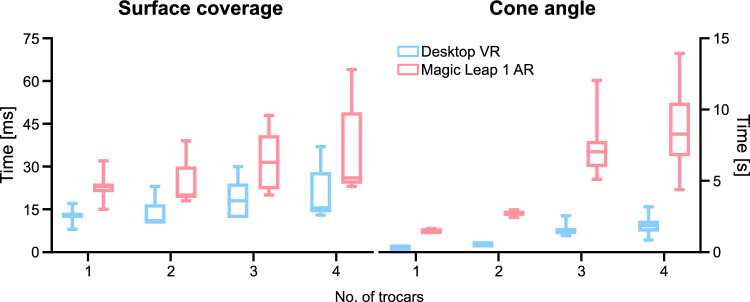
Calculation times of our algorithms based on the surface coverage metric (left) and cone angle (right) for two devices: Desktop PC (blue) and Magic Leap 1 (pink). Data presented for 4 different trocar configurations. *Less is better.*

## Results

Figures in this section present calculation times for several use cases, focusing on using the desktop VR application and standalone AR solution deployed to ML1. In each case, we ran the algorithm 240 times to find the optimal pose based on the surface coverage and the cone angle for four different trocar configurations, as described in “Performance testing” section. The statistical analysis was performed using Two-Way ANOVA and uncorrected Fisher’s LSD multiple comparisons test.

Figure 5 shows run times when calculating the optimal pose on a desktop VR setup and in standalone mode on ML1 for both metrics. Below, we reported the data as mean ± SD. Starting with a single trocar and based on reduced workspace data, the PC takes $12.78 \pm {2.83}{ms}$, while the ML1 finishes in $23.38 \pm 5.24$. The cone angle for the same case was calculated in $311.15 \pm 16.88$ and $1488.82 \pm {69.10}{ms}$ for the desktop and AR, respectively. Adding a second trocar increases slightly the time needed for calculation on the desktop ($14.34 \pm {5.04}{ms}$), while on the AR device, the recorded time rose marginally to $24.94 \pm 8.21$. We observed a much higher running times for cone calculations: $540.40 \pm 31.86$ milliseconds - VR and $2719.36 \pm 138.46$ - AR. In the scenario where tree ports are used, the time increased on both devices. The process took $18.45 \pm 6.06$ and $31.33 \pm 9.68$ milliseconds on average for the desktop VR and the AR standalone application, respectively. Again, we see another bump in the time needed to find the solution based on cone angle: $1577.86 \pm 456.42$ on the PC, and $7373.45 \pm {1982.08}{ms}$ on ML1. The final run with four trocars unsurprisingly prolonged the calculations to $21.31 \pm {9.32}{ms}$ for the desktop and $36.66 \pm {15.48}{ms}$ for the AR headset. The cone angle calculation in this scenarios took $1892.35 \pm {669.22}{ms}$ and $8601.64 \pm {2938.43}{ms}$ on the VR and AR setups, respectively.

**Fig. 6:**
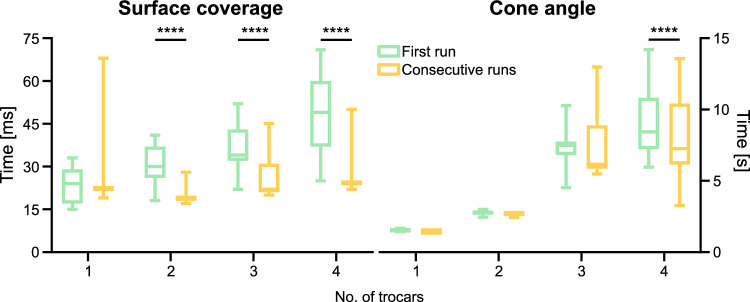
A comparison between running our algorithms for the first time (green) and in consecutive runs (yellow) on Magic Leap 1 AR headset. Left side: optimal pose calculation based on surface coverage; right side: based on cone angle. *Less is better.*

Figure 6 provides a comparison of processing times on the Magic Leap 1 headset, focusing on the initial calculation of the algorithm and its subsequent runs when a random trocar is repositioned.

Two-way ANOVA reports a significant difference between groups (P<0.0001, F = 162.9 and P<0.0001, F = 8.411 for surface coverage and cone angle, respectively). The values reported here are mean values from 240 trials ± SD in milliseconds. In the scenario where we simulated SPL, the function returned the optimal robot/patient pose based on surface coverage after $23.84 \pm {6.14}{ms}$ in the first run and $22.91 \pm {4.11}{ms}$ in the following triggered trials (not a significant difference). With two port location taken into account, the consecutive computations took significantly shorter, drop from $30.83 \pm {7.96}{ms}$ to $19.06 \pm {1.46}{ms}$. Again, with three trocars, the algorithm in the second run performed better—$36.83 \pm 8.83$ milliseconds in the first run and $25.81 \pm 6.97$ in the following ones. The last scenario based on the surface coverage metric reported a significant reduction in running times - $48.02 \pm {14.41}{ms}$ to $25.30 \pm {3.65}{ms}$.

In an SPL scenario, the cone angle estimation on the AR headset takes $1527.53 \pm 79.67$ milliseconds on the first run and $1450.11 \pm 14.58$ in consecutive runs. The introduction of another port approximately doubled the time needed to $2760.26 \pm 149.52$ and $2678.46 \pm 112.75$ for the first and the following trials, respectively. In the next case, the algorithm calculated the optimal solution for the first time in $7341.09 \pm 11353.04$ milliseconds on average, while the consecutive calculations took $7405.81 \pm 2457.81$. Finally, the last scenario with four port locations took initially $9078.18 \pm {2651.80}{ms}$, then to drop to $8125.10 \pm {3133.17}{ms}$. Only in the last tested scenario, the consecutive trials took significantly shorter to complete the task (P<0.0001).

## Discussion

Introducing new robotic systems in the OR will force the surgical staff to adapt to new hardware quickly. Understanding the capabilities and limitations of the system is essential to a successful surgical intervention. The process can be facilitated through augmented reality visualization systems helping the users to comprehend the device, e.g., the reachability of the robotic arm. Additionally, a proper patient-robot arrangement plays a crucial role in a smooth and uninterrupted course of the surgery, which often requires preoperative planning. We believe that our system is well-suited to assist in these processes.

Previously, we demonstrated how our AR system could improve the users’ performance in patient positioning tasks in single-port laparoscopy. With multiple ways of presenting rotational and positional workspace information in the OR, we could enhance the reachability at the trocar location across all testers. That said, none of the users could consistently reach the levels of the computer algorithm. In addition, the task involving multiple trocars is even more demanding mentally and challenging for the user. Therefore in this work, we proposed an automatic patient/robot positioning system in the OR using robot workspace information based on our previous implementation.

With software like Specto, the surgeon can examine the patient’s imaging data and, knowing the robotic system’s characteristics, can prepare accordingly for an upcoming procedure. This can minimize the risk of complications for the patient and reduce the possibility of repositioning and redock the robot in the middle of surgery. With our system, the user gets the most optimal robot-patient configuration in a matter of seconds or milliseconds, depending on which metric is selected. This significantly reduces the time and alleviates the mental burden of doing this task manually.

Additionally, based on the performance tests, our system can be deployed directly to the OR using AR glasses with the same functionality as the VR version. Our application running on a Magic Leap 1 headset can guide the personnel in positioning the robot in the OR and, if necessary, rerun the calculations with repositioned trocars. While the computations take much longer in the AR application, it is still in an acceptable range, thus not causing delays in the setup stage of the surgery.

The adoption of AR devices in the OR can be challenging, and it will require proper planning and collaboration between surgeons, healthcare providers, and technology experts. We plan to focus on the usability aspect of the system to ensure seamless integration into surgical workflow. An improvement can be achieved by, among other things, integrating the navigation system used by the robotic platform, which would allow for a quick and more accurate patient-robot positioning. Additionally, in the current version, all the interactions are performed with controllers; we intend to implement a gesture interaction system that would allow the use of the system even during surgery.

## Conclusions

Building upon the previous implementation, we expanded the number of supported ports and changed the system’s overall design to facilitate automatic optimal pose calculation. The changes and optimizations we implemented significantly boosted the performance, offering the best possible solution in seconds in every tested scenario.

With the increasing number of new robotic systems in practice, our solution can speed up the deployment of such systems by making it easier for the staff to get acquainted with them, as demonstrated in the previous work. At the same time, we are removing some mental strain and saving time for staff preparing the OR, suggesting an optimal patient-robot configuration.

Our results indicate that AR devices have the potential to improve the efficiency and accuracy of surgical procedures; however, they still require a lot of investment and work both in the field of software development as well as in hardware design.
